# Preliminary Effects of American Elderberry Juice on Cognitive Functioning in Mild Cognitive Impairment Patients: A Secondary Analysis of Cognitive Composite Scores in a Randomized Clinical Trial

**DOI:** 10.3390/antiox14020131

**Published:** 2025-01-23

**Authors:** Madison Musich, Ashley F. Curtis, Bradley J. Ferguson, David Drysdale, Andrew L. Thomas, C. Michael Greenlief, Joel I. Shenker, D. Q. Beversdorf

**Affiliations:** 1 Department of Psychological Sciences, University of Missouri, Columbia, MO 65201, USA; mmusich@missouri.edu; 2 College of Nursing, University of South Florida, Tampa Bay, FL 33620, USA; ashleycurtis@usf.edu; 3 Department of Neurology, University of Missouri, Columbia, MO 65201, USA; fergusonbj@health.missouri.edu (B.J.F.); shenkerj@health.missouri.edu (J.I.S.); 4 Departments of Biology and Chemistry, St. Ambrose University, Davenport, IA 52803, USA; drysdaledavidr@sau.edu; 5 School of Medicine, University of Missouri-Kansas City, Kansas City, MO 64110, USA; 6 Division of Plant Science and Technology, Southwest Research Extension and Education Center, University of Missouri, Mt. Vernon, MO 65201, USA; thomasal@missouri.edu; 7 Department of Chemistry, University of Missouri, Columbia, MO 65201, USA; greenliefm@missouri.edu; 8 Department of Radiology, University of Missouri, Columbia, MO 65201, USA

**Keywords:** mild cognitive impairment, cognitive flexibility, antioxidants, elderberry, *Sambucus*

## Abstract

Previous work examining dietary interventions high in polyphenols (i.e., antioxidant/anti-inflammatory properties) has shown cognitive benefits. In a prior investigation examining American elderberry juice consumption with inflammation and cognition in mild cognitive impairment (MCI), we found a trend toward better visuospatial construct flexibility in MCI patients who consumed elderberry relative to the placebo control. This study aims to further examine the preliminary effects of American elderberry juice on the cognitive domains in MCI using cognitive composite scores. MCI patients (*N* = 24; *M*_age_ = 76.33 ± 6.95) received elderberry (*n* = 11) or placebo (*n* = 13) juice for 6 months and completed cognitive tasks targeting memory, visuospatial ability, and cognitive flexibility at the baseline, 3 months, and 6 months. For the composite *z*-scores calculated for global cognition and each domain, multilevel models and Kenward–Roger post hoc tests examined the interaction between condition (elderberry/placebo) and time (baseline/3 months/6 months). The findings showed a significant interaction between global cognitive flexibility (*p* = 0.049) and elderberry juice (not the placebo) trending toward a significant decrease (better) in composite cognitive flexibility latency scores from the baseline (*M* = 29.89, *SE* = 18.12) to 6 months (*M* = 18.57, *SE* = 9.68). Preliminary findings suggest elderberry juice may provide overall cognitive flexibility benefits in MCI. These promising results provide support for prospective investigations examining the potential underlying mechanisms of elderberries that may provide cognitive benefits in MCI, possibly due to anti-inflammatory effects.

## 1. Introduction

Mild cognitive impairment (MCI) is characterized as the transition between cognitive states of normal cognitive aging and dementia [1] and is considered as amnestic (cognitive impairment in memory is prominent) or non-amnestic (cognitive impairment in a non-memory cognitive domain is prominent) [2]. Although there are no current effective pharmacological treatments for MCI patients [2], antioxidants and anti-inflammatory drugs have been proposed as potential agents for MCI treatment [3]. Polyphenols and anthocyanins contain significant antioxidant properties [4,5,6], which can counteract oxidative stress (imbalance between reactive oxygen species and antioxidants) and inflammation [7,8,9], therefore providing neuroprotective benefits against neurodegeneration [9] by combatting mitochondrial oxidative stress [10]. Previous work in our lab investigated the impact of a 6-month elderberry (an anthocyanin dietary supplement that contains high anti-inflammatory and antioxidant properties [11,12]) intervention on peripheral blood-based inflammatory markers in older adults with MCI [13]. The findings showed that overall low-grade peripheral inflammatory markers were reduced after 6 months. Preliminary secondary analyses for changes in cognitive performance showed a trend toward improved visuospatial cognitive flexibility in older adults with MCI who consumed elderberry juice relative to the placebo control. We did not observe (or found only those trending toward) significant associations across 6 months between elderberry juice consumption and various cognitive tests for global cognition (Mini-Mental State Examination), language (Boston Naming Test), verbal memory (Hopkins Verbal Learning Test), visuospatial memory and construction abilities (Rey–Osterrieth Complex Figure Test), and cognitive flexibility (Anagrams and Visuospatial Problem Solving). Further examining the impact of supplemental elderberry juice consumption across cognitive domains measured as composite scores may elucidate the potential cognitive benefits of elderberry consumption in older adults with MCI.

Polyphenols and anthocyanins have been suggested to combat cognitive decline [14]. For instance, in cognitively healthy older adults, a polyphenol-rich diet intervention showed improvements in working memory [15] and memory [16,17] relative to controls. However, the work that exists for investigating the impact of a polyphenol- and anthocyanin-rich diet intervention in older adults with MCI has shown improved visual spatial learning, processing speed, self-reported executive functioning [18], and a trend for better visuospatial cognitive flexibility [13]. Previous investigations have primarily examined each cognitive assessment, inflating the Type 1 error rate [19]. Further investigating the impact of a dietary anthocyanin-rich elderberry juice intervention on cognitive composite scores in older adults with MCI may better detect subtle changes in cognitive performance, while limiting the number of statistical tests required [20] and lowering the Type I error rate [19,21]. Cognitive composite scores show better statistical power and efficiency in clinical populations [20], such as preclinical Alzheimer’s disease populations [19]. Specifically, these analyses can increase the measurement precision for assessing cognitive abilities [20] and are more powerful in detecting cognitive changes relative to the constituent components in the cognitive measures [20,22]. Standardized (i.e., *z*-scores) cognitive composite scores can further determine treatment effects to a greater degree relative to non-transformed multivariate statistics [21]. Therefore, examining the impact of elderberry juice consumption and cognitive composite scores in older adults with MCI may detect the treatment effects to a greater degree on the specific cognitive domains relative to the constituent components, as previously investigated [13].

The present study aimed to further examine the preliminary association of American elderberry (*Sambucus nigra* subsp. *canadensis*) juice consumption (placebo control vs. elderberry) with various specific cognitive domains in older adults with MCI across 6 months in a secondary analysis of a randomized clinical trial [13]. We hypothesized that older adults with MCI who consume elderberry juice will show better cognitive functioning across various domains (composite scores for global cognition, visuospatial construct ability, cognitive flexibility for accuracy and speed, and memory) after 6 months compared to older adults with MCI consuming the placebo-control juice.

## 2. Materials and Methods

### 2.1. Participants

The present study was a secondary analysis of data from a randomized, double-blinded, placebo-controlled clinical trial (NCT02414607; PI: Beversdorf) that investigated the feasibility and preliminary efficacy of American elderberry juice in MCI patients, which revealed an effect on proteomic inflammatory markers [13]. Physician referrals (D.Q.B., J.S) from a Memory Disorder Clinic in Columbia, MO, were used to recruit participants with an amnesic MCI diagnosis. Participant inclusion criteria included (1) aged 50+ years, (2) currently diagnosed with MCI, (3) a score of 0.5 on the Clinical Dementia Rating Scale (CDR, administered by study physicians D.Q.B. or J.S.) to indicate very mild dementia, (4) a Mini-Mental State Examination (MMSE [23]) score of 24+/30, (5) self-reported no known history of sensitivity or allergy to elderberry products, and (6) no presence of a medical condition that would impair the participant’s ability to complete the study (confirmed with the clinical experience of the investigators). In addition to the inclusion criteria of participants having a CDR of 0.05, participants were diagnosed with MCI from board-certified specialists in Behavioral Neurology and Neuropsychiatry. Participant exclusion criteria included (1) self-reported known history of allergy to honeysuckle (closely related to elderberry), (2) a current diabetes diagnosis, (3) presence of a bleeding disorder, (4) currently pregnant, (5) current changes in medications that may impact cognitive performance, (6) current comorbid medical or psychiatric condition that would impact the ability to complete study procedures, and (7) the presence of other neurodegenerative diseases. The University of Missouri Institutional Review Board approved all study procedures.

### 2.2. Study Design and Procedure

As previously reported [13], this randomized, double-blinded, placebo-controlled trial consisted of three study visits at the baseline, 3 months, and 6 months. Participants were randomized to either the placebo-control juice or elderberry juice intervention condition and consumed 5 mL of juice orally three times a day for 6 months. At each study visit, participants received an approximate supply of 3 months of elderberry juice and were instructed to store the juice in a refrigerator before consumption. Before the visits distributing the juice, the supply of juice for both conditions was stored in a refrigerator located in our lab. The *Sambucus nigra* subsp. *canadensis* (North American Elderberry) strain of the elderberry juice was 100% commercially available and stabilized with citric acid from River Hills Harvest (Hartsburg, MO, USA; see [13] for further details), consisting of 15.9 mg of cyanidin-3-glucoside (the main anthocyanin in elderberry) per 5 mL of elderberry juice. This dosage is similar to the previous well-tolerated doses that examined the effects of elderberry juice on lipoproteins [11]. The placebo-control juice contained flavored and colored water resembling elderberry with no nutritional value. At each study visit, participants received the required amount of juice to last until the next study visit. Participants were instructed to document their juice consumption in a daily juice log, as well as save the empty juice containers to confirm the juice consumption that was documented in the juice log. Participants who consumed <75% of the scheduled dose for each study visit were excluded. Participants also completed various cognitive tasks at each study visit with alternative forms for each measure (see Section 2.3).

### 2.3. Measures

Full details of the cognitive measures are provided in the main outcomes paper [13]. For the purpose of the present study, brief descriptions of the cognitive tasks are provided.

#### 2.3.1. Mini-Mental State Exam

The Mini-Mental State Examination (MMSE) is a brief (~5 min) assessment of mental status/global cognitive functioning [23]. The MMSE was used to determine whether global cognitive functioning is not beyond MCI with a cutoff score of 24+/30.

#### 2.3.2. Hopkins Verbal Learning Test

The Hopkins Verbal Learning Test (HVLT) assesses verbal learning and memory, and consists of two parts measuring verbal recall and recognition [24]. HVLT full methods are discussed in detail elsewhere [13]. First, for the verbal recall part, participants were orally presented with a word list (12 words) over 3 trials and were instructed to recall the list of words once again after a 20 min delay. During the final recognition part, 24 words were presented, including the 12 original words (i.e., true positive), 6 new related distractor words (i.e., false positive-related), and 6 new unrelated distractor words (i.e., false positive-unrelated). Participants indicated whether each word was presented in the original word list. The following outcomes of interest were computed: HVLT-Free Recall (total number of words accurately recalled from trials 1–3 on the HVLT recall portion), HVLT-Delayed Free Recall (total number of words accurately recalled), HVLT-Recognition Number (#) Hits (total number of correct true positive words recognized, higher values indicating better memory), HVLT-Recognition False Alarm (FA)-related (total number of false positive-related words recognized, lower values indicating better memory), HVLT-Recognition FA-unrelated (total number of false positive-unrelated words recognized, lower values indicating better memory), and HVLT-Discrimination Index (difference between HVLT-Recognition # Hits and HVLT-Recognition FA grand total, higher values indicating better memory).

#### 2.3.3. Rey–Osterrieth Complex Figure Test

The Rey–Osterrieth Complex Figure Test (REY-CFT) measures visuospatial constructional ability and visual memory [25]. Participants are instructed to immediately copy a complex image on a separate blank sheet of paper as accurately as possible and to redraw the complex image from memory after 30 min. Higher REY-CFT Delayed Copy Total scores indicate better visuospatial memory, with a total possible score of 36.

#### 2.3.4. Anagrams

The anagram problem solving tasks (i.e., Anagrams) measures verbal cognitive flexibility and convergent creativity [26,27]. A set of 20 anagrams [14 five-letter (mildly challenging) and six seven-letter (difficult) anagrams] were individually presented to participants, and they were instructed to unscramble each anagram within 120 s. The average total number of correct anagrams completed (i.e., Anagrams-Total Correct) was computed, with higher scores indicating an increased accuracy of verbal cognitive flexibility. The average latency (via ms) to solution (i.e., Anagrams-Latency Total) with failed solutions recorded as 120 s was computed, with lower scores indicating a better speed of verbal cognitive flexibility.

#### 2.3.5. Visuospatial Problem Solving

The Visuospatial Problem Solving (VPS) task is adapted from the matchstick problems in previous work assessing cognitive flexibility and convergent creativity [28,29]. A total of six line/matchstick problems were presented to participants, and they were instructed to either mentally move or remove a specified set number of lines/matchsticks to construct the specified configuration within four minutes [30]. The average total number of correct VPS problems completed (i.e., VPS-Total Correct) was computed, with higher scores indicating an increased accuracy of cognitive flexibility and convergent creativity. The average solution latency (i.e., VPS-Mean Latency Correct Score via ms) was computed, with lower scores indicating a better speed of cognitive flexibility and convergent creativity.

### 2.4. Statistical Analysis

Scores from each cognitive measure were computed as *z*-scores via the mean and standard deviation for each group (placebo/condition), as recommended by [22]. *Z*-scores were then averaged across participants for each group to calculate the composite scores to assess cognitive domains. HVLT-FA unrelated and related subscores were recoded to ensure all HVLT subscores were interpreted in the same direction (i.e., higher scores indicating better performance). For global cognition, the total *z*-scores from each cognitive measure were averaged. A scale transformation on the *z*-score composite scores was also computed for the cognitive domains of visuospatial construct ability (average *z*-scores for Rey CFT Copy Total and Delayed Copy Total scores; higher scores indicate better visuospatial construction performance), cognitive flexibility for accuracy (total number correct) (average *z*-scores for Anagrams and VPS Total Correct scores; higher scores indicate better cognitive flexibility performance), speed (latency) (average *z*-scores for Anagrams and VPS latency (measured via ms) scores, lower scores indicate better cognitive flexibility performance) and rate of correct score (RCS, correct scores were divided by the sum of the reaction time, with higher scores indicating better cognitive flexibility efficiency [31]), and memory (average *z*-scores for HVLT subscores and the Rey CFT Delayed Copy Total subscore, higher scores indicating better memory performance). Comprehensive composite scores with subdomains for visuospatial construct ability and memory were collated, given that previous work [32] showed that a comprehensive memory composite score outperformed memory subdomain composite scores to detect change and predict conversion from MCI to Alzheimer’s disease.

A secondary analysis from a randomized clinical trial [13] used separate multilevel model (MLM) analyses (via *lmer* [33]) conducted in R [34] to test the fixed effects of a condition (placebo-control, elderberry; placebo-control coded as the reference level), time (baseline, 3-month follow-up, 6-month follow-up), and the interaction between the conditions and the four outcome variables of the cognitive composite scores for global cognition, visuospatial construct ability, cognitive flexibility, and memory. In each MLM, participants were nested within a condition. Restricted maximum likelihood estimation was used in the MLM analyses to reduce the Type I error inflation [35,36], given the present study’s small and unbalanced sample size. Alpha-level *p* < 0.05 was used for all the MLM analyses. In the instance of a significant fixed effect, Kenward–Roger post hoc least square means pairwise comparisons (via *dfflsmeans* [37]) further evaluated the significant (*p*s < 0.05) and trending (*p*s < 0.10) associations between condition and time fixed effects and the composite scores for each cognitive outcome variable. In the instance that our visuospatial construct ability and/or memory composite scores were statistically significant, exploratory MLM analyses with subdomain composite scores were evaluated. In MLM analyses, η^2^ was used to describe the effect sizes for significant interactive terms [small (η^2^ = 0.01), medium (η^2^ = 0.06), and large (η^2^ = 0.14); [38,39]], while Hedge’s g (*g_av_*) was calculated to describe the post hoc least square mean pairwise comparisons [small (*g_av_* = 0.20), medium (*g_av_* = 0.50), and large (*g_av_* = 0.80); [38]]. Given that the present study is a pilot investigation, family-wise error corrections were not implemented, and we accept the false-positive risk.

## 3. Results

### 3.1. Participant Characteristics

**Figure 1:**
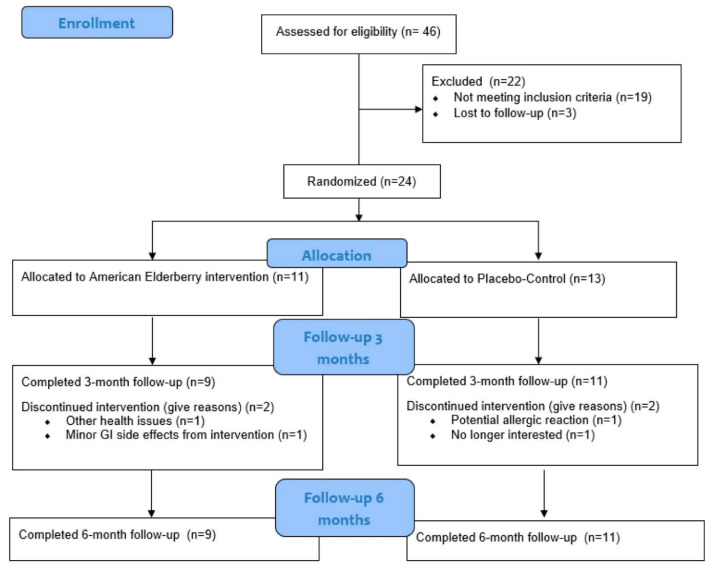
Participant Consolidated Standards of Reporting Trials (CONSORT) Diagram. Note. Adapted from [13].

A total of 13 participants were randomized to the placebo-control condition (*M*_age_ = 76.23, *SD*_age_ = 7.23, 7 male/6 female), and a total of 11 participants were randomized to the elderberry condition (*M*_age_ = 76.45, *SD*_age_ = 8.47, 3 male/8 female), with all participants reporting their race as White and ethnicity as non-Hispanic. Of the 24 participants who were randomized in the present study, two participants in the elderberry condition and two in the placebo-control condition did not complete the 3-month follow-up study visits due to gastrointestinal issues and health issues, as outlined in the Consolidated Standards of Reporting Trials (CONSORT) in Figure 1, as documented in the initial study [13]. All participants completed all measures and were included in the analyses. The complete participant demographics for the present study can be found elsewhere [13]. Table 1 shows the descriptive values for the composite score of the cognition variables (*M*s and *SD*s for cognition subscores can be found elsewhere [13]). At the baseline, differences between the elderberry and placebo-control conditions were evaluated by independent *t*-tests for continuous variables and chi-square tests for categorical variables at alpha-level 0.05. Differences between conditions were not observed for the demographic (*p*s > 0.05) and cognition (see Table 1) variables at the baseline.

### 3.2. MLM Results

**Figure 2:**
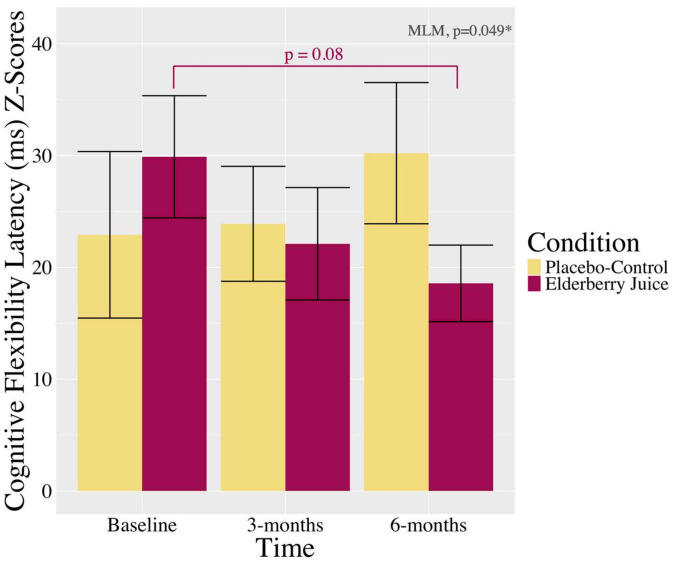
Changes in the composite scores of cognitive flexibility (mean latency for correct trials) across 6 months in the American elderberry condition compared to the placebo-control condition in patients with MCI. Note. Anagrams and Visuospatial Problem Solving task latency (ms) subscores were computed as z-scores, and the average z-score from the task was calculated as the composite score for cognitive flexibility. Lower global cognitive flexibility latency scores indicate better performance. There was a significant interaction between condition and time on cognitive flexibility latency composite scores (*p* = 0.049), such that those with MCI who consumed the elderberry juice showed a trend toward a significant decrease (better) between the baseline and 6-month follow-up study visits for global cognitive flexibility. There was no observed trending or significance associations for other time points for those with MCI consuming the elderberry juice or for those who consumed the placebo-control juice across time. Error bars = *SE*; ms = millisecond; MCI = mild cognitive impairment. * *p* < 0.05.

Table 2 shows the full MLM fixed effects for each cognition composite score outcome variable. The interaction between condition and time on the composite score for cognitive flexibility was statistically significant (*p* = 0.049, η^2^ = 0.077; see Figure 2). Post hoc pairwise comparison tests show that in the elderberry condition, cognitive flexibility scores were trending toward a significant decrease (better) between the baseline (*M* = 29.89, *SD* = 18.12) and 6-month follow-up (*M* = 18.57, *SD* = 9.68) study visits [*t*(39.1) = 1.80, *p* = 0.08, 95% CI: (−1.62, 28.29), *g_av_* = 0.72 (medium effect size [38])], while no trending or significant differences were observed between the baseline and 3 months (*p* = 0.22) or between 3 months and 6 months (*p* = 0.55) follow-up study visits. Post hoc pairwise comparison tests did not show significant or trending associations with composite cognitive flexibility scores for the placebo-control condition (*p*s > 0.05). The interactions between condition and time were not significantly associated with the composite scores for global cognition, visuospatial construct ability, cognitive flexibility total number correct, cognitive flexibility RCS, and memory (*p*s > 0.05; see Table 2). Similarly, significant or trending independent associations of condition and time were not observed for the composite scores of each cognition domain (*p*s > 0.05; see Table 2).

## 4. Discussion

This secondary analysis of a randomized clinical trial [13] further examined the effect of American elderberry juice consumption on various cognitive domain composite scores across 6 months in older adults with MCI. The findings showed that elderberry juice consumption led to a trending improvement in the domain of cognitive flexibility for speed (not accuracy) after 6 months in older adults with MCI, whereas no such improvement was observed for the placebo-control juice, with a significant time x treatment condition interaction effect.

Partially consistent with our hypothesis, we observed a specific cognitive domain score improvement of speed for cognitive flexibility (i.e., cognitive flexibility latency scores) at 6 months following American elderberry juice consumption in older adults with MCI compared to the placebo-control juice condition. Our finding extended our prior work [13], showing that American elderberry juice may more broadly impact overall cognitive flexibility. This is in partial agreement with previous investigations that found a diet intervention rich in polyphenols and anthocyanins showed better self-reported executive functioning [18]. It is possible that the *z*-transformed cognitive composite scores in the present study provide statistical advantages to detect cognitive changes [20,22] and treatment effects [21]. Specifically, composite scores may reduce the intra-individual variability and reflect more accurate changes in cognition across time [40] relative to constituent components, as shown in our previous investigation [13].

Although we observed a significant interaction between the cognitive flexibility composite score and speed, we did not observe such relationships for the cognitive flexibility composite scores of accuracy (i.e., cognitive flexibility total scores) and RCS. Evaluating accuracy, speed, and RCS permits a comprehensive understanding of cognitive performance [41] for cognitive flexibility. It is possible that we did not observe associations between condition (placebo-control, elderberry juice) and time on cognitive flexibility scores for accuracy and RCS, given that participants in the present study did not exhibit errors on Anagrams and VPS measures for cognitively flexibility. Rather, participants generally failed to complete the measure due to the time constraints, which is taken into account when computing the latency scores for Anagrams and VPS.

It is also possible anti-inflammatory properties in elderberries may contribute to cognitive benefits relating to cognitive flexibility for speed (not for accuracy or RCS). Elevated inflammatory levels are linked with cognitive decline [42,43] and Alzheimer’s disease pathology [44]. Decreased inflammation and oxidative stress states may further attenuate mitochondrial oxidative stress levels [45] and potentially provide cognitive benefits related to cognitive flexibility in older adults with MCI. This is important given that our previous findings showed that generally low-grade peripheral blood-based inflammation was reduced after 6 months of consuming elderberry juice in older adults with MCI [13]. Antioxidant and anti-inflammatory properties in elderberries [11,12] may decrease inflammation and oxidative stress state [7,8,9] burden in the frontal and parietal areas [45] that are important for cognitive flexibility abilities [46]. Polyphenols have also been suggested to be neuroprotective [47], such as neural architecture protection [48], and stimulating neurogenesis in brain regions associated with working memory (involved with cognitive flexibility) and learning [49]. Therefore, it is possible that the high level of antioxidant properties that are found in elderberries may reduce oxidative stress levels in frontal and parietal regions [45], which may contribute to improved overall cognitive flexibility performance, for which latency is a more sensitive measure.

### 4.1. Clinical Implications

The present preliminary study has several potential clinical implications. First, given the inconsistencies across a variety of cognitive assessments are used for evaluating MCI and dementia [50], computing composite scores for cognitive domains rather than constituent parts may increase cognitive measurement precision [20], increase detection for changes in cognition, and may be more powerful to evaluate prodromal phases of Alzheimer’s dementia [19]. Second, elderberry consumption may have clinical utility as a non-invasive nutritional supplement with minimal side effects to prevent the further cognitive decline in MCI populations. Currently, there are no effective pharmacological treatments for MCI patients [2], and non-pharmacological treatments have been proposed as potential agents in MCI treatment [3]. Elderberry antioxidant and anti-inflammatory properties [11,12] may support cognitive health in aging populations [51] through potentially attenuating the potential underlying mechanisms contributing to cognitive decline, such as inflammation [44,52,53]. Importantly, the implementation of elderberry juice as a non-invasive nutritional supplement may reduce the risk of MCI patients developing Alzheimer’s disease. For instance, in a previous animal model, an antioxidant treatment prevented amyloid-beta accumulation in the parietal and entorhinal cortices (i.e., regions important for cognitive flexibility abilities; [54]). Of importance, amyloid-beta accumulation in this region is linked with the transition of MCI to Alzheimer’s disease [55]. Therefore, the present study’s preliminary findings that elderberry juice may provide cognitive benefits in MCI patients are promising, but larger prospective investigations are warranted to further understand the clinical utility of elderberry juice in MCI populations.

### 4.2. Limitations and Future Directions

In addition to the limitations in the main outcomes paper [13], there are several additional potential limitations for the present study that should be considered. First, the sample size for this preliminary investigation was small. Future investigations should examine the association between elderberries and cognition in a larger sample size with balanced conditions (elderberry vs. control) and a balanced distribution between males and females. Additionally, a simple randomization was used to randomize participants into elderberry or placebo-control conditions in the present study, which resulted in unequal groups. Future investigations are encouraged to utilize stratified random sampling for equal groups. Second, the time frame of elderberry consumption (6 months) in the present study did not include longer-term follow-up study visits (i.e., 1 year). Although the present study observed a significant association between elderberry juice consumption and cognitive flexibility ability after 6 months, and this time frame is in line with previous investigations [13,18,56], it is important to further investigate this effect across longer time points. For instance, one review examining studies with anthocyanin-rich food interventions showed that anthocyanins tend to first show cognitive benefits relating to verbal learning and memory, while the longer-term consumption of anthocyanin-rich food can provide cognitive benefits relating to working memory and attention [57]. It is possible that a longer-term intervention of elderberry juice may show changes in cognition in more than one cognitive domain. Further investigating this relationship across a longer time frame may inform the timing of antioxidant and anti-inflammatory property benefits from elderberries across potentially several cognitive domains. Third, although the present study examined the association of an elderberry juice intervention on cognitive functioning in older adults with MCI, it is important to further investigate whether these promising preliminary effects potentially mitigate the conversion of MCI to dementia of the Alzheimer type. Lastly, the present study did not assess participants’ typical food diets or whether participants consumed foods that may have similar properties and effects to elderberry juice. This is of particular importance given that diets such as the Mediterranean diet are rich in antioxidant [58] and anti-inflammatory compounds [59] that share antioxidant and anti-inflammatory properties with elderberry juice [11,12]. Future investigations should examine the potential influence of existing diets and food consumption that may have similar properties to elderberry juice to further delineate the potential underlying mechanisms of elderberries that may provide cognitive benefits in MCI. Future investigations are also encouraged to consider changes in existing diets as an exclusionary criterion to elucidate the potential influence of elderberry juice consumption on cognitive functioning relating to cognitive flexibility.

## 5. Conclusions

The present secondary analysis of a double-blind, randomized clinical trial [13] further supports the impact of American elderberry juice consumption on cognitive functioning in older adults with MCI. The findings showed that American elderberry consumption may broadly impact cognitive flexibility (i.e., an executive function) in older adults with MCI relative to the placebo control. Prospective investigations examining the impact of elderberry consumption on cognitive functioning over longer time frames are warranted to further point to the clinical utility of elderberry for mitigating further cognitive decline.

## Figures and Tables

**Table 1:** Descriptive Statistics for Cognitive Composite Score Variables of Older Adults with Mild Cognitive Impairment.

	Placebo-Control Condition (*N* = 13)						Elderberry Condition (*N* = 11)						Baseline Condition Comparisons
	Baseline		3-Month Follow-Up		6-Month Follow-Up		Baseline		3-Month Follow-Up		6-Month Follow-Up		
**Variables**	***M***	***SD***	***M***	***SD***	***M***	***SD***	***M***	***SD***	***M***	***SD***	***M***	***SD***	***p***
Global Cognition	12.73	2.03	13.27	1.55	12.86	2.43	13.05	2.27	12.68	2.25	12.82	1.88	0.72
Visuospatial Construct Ability	13.34	5.59	13.43	4.58	12.56	6.26	14.26	4.43	13.92	5.02	13.40	4.33	0.67
Cognitive Flexibility Total Correct	2.86	2.37	4.79	2.55	4.43	2.62	4.84	3.97	4.09	4.51	4.91	3.72	0.14
Cognitive Flexibility Latency	22.91	26.85	23.89	17.05	30.21	20.94	29.89	18.12	22.11	15.08	18.57	9.68	0.47
Cognitive Flexibility RCS	0.15	0.21	0.22	0.11	0.18	0.19	0.14	0.15	0.23	0.43	0.37	0.26	0.85
Memory	1.98	2.04	2.10	1.69	1.62	1.75	1.20	2.16	1.24	1.72	0.62	1.78	0.38

Note. The *M*s and *SD*s for cognition subscores can be found elsewhere [13]. An independent *t*-test evaluated condition differences between the placebo-control and elderberry groups. HVLT, REY-CFT, Anagrams, and VPS subscores, as presented, were computed as a *z*-score to calculate composite scores for assessing the global scores for each cognitive domain. The *z*-scores for HVLT-FA unrelated and related subscores were scaled to ensure that all HVLT subscores were interpreted in the same direction, such as higher scores indicating better performance. The total *z*-scores from each cognitive measure were averaged to calculate a global cognition score. Visuospatial construct ability was calculated as the average *z*-scores from REY-Copy Total Correct and REY-Delayed Copy Total Correct subscores. Cognitive flexibility total correct for measuring accuracy was calculated as the average *z*-scores from the total correct scores for Anagrams and VPS Mean Latency Total subscores. Cognitive flexibility latency for measuring speed was calculated as the average *z*-scores from Anagrams Mean Latency (ms) and VPS Mean Latency Total (ms) subscores. Cognitive flexibility RCS was calculated using the total correct z-score divided by the sum of the latency score for Anagrams and VPS [31]. Memory was calculated as the average *z*-scores from the HVLT-subscores and REY-Delayed Copy Total Correct subscore. HVLT = Hopkins Verbal Learning Test; REY-CFT = Rey–Osterrieth Complex Figure Test; VPS = Visuospatial Problem Solving; RCS = Rate Correct Score; ms = milliseconds; FA = False Alarm.

**Table 2:** Multilevel Modeling Results of Fixed-Effect Associations Between Condition, Time, and their Interaction on Various Cognitive Domains in Older Adults with Mild Cognitive Impairment (*N* = 24).

	Condition				Time				Condition × Time				
**Variable**	***b***	***SE***	***t***	***p***	***b***	***SE***	***t***	***p***	***b***	***SE***	***t***	***p***	**η^2^**
Global Cognition	0.20	1.01	0.20	0.85	−0.01	0.23	−0.04	0.97	−0.04	0.35	−0.11	0.91	0.001
Visuospatial Construct Ability	0.95	2.50	0.38	0.71	−0.02	0.56	−0.03	0.98	−0.15	0.81	−0.18	0.86	0.001
Cognitive Flexibility Total Correct	2.11	1.96	1.073	0.29	0.55	0.29	1.89	.07	−0.62	0.44	−1.43	0.16	0.02
Cognitive Flexibility Latency	17.06	11.003	1.55	0.99	2.91	3.15	0.92	0.36	−9.70	4.79	−2.03	0.049 *	0.077
Cognitive Flexibility RCS	−0.15	0.15	−1.01	0.32	0.01	0.04	0.27	0.79	0.11	0.07	1.66	0.11	0.04
Memory	−0.67	2.33	−0.29	0.77	−0.13	0.20	−0.63	0.54	−0.14	0.30	−0.47	0.64	0.001

Note. HVLT, REY-CFT, Anagrams, and VPS subscores, as presented, were computed as a *z*-score to calculate global scores (i.e., composite scores) for the cognitive domains. The HVLT-FA unrelated and related subscores were scaled to ensure that all HVLT subscores were interpreted in the same direction, such as higher scores indicating better performance. The total *z*-scores from each cognitive measure were averaged to calculate a global cognition score. Visuospatial construct ability was calculated as the average *z*-score from REY-Copy Total Correct and REY-Delayed Copy Total Correct subscores. Cognitive flexibility total correct was calculated as the average *z*-score from the total correct scores for Anagrams and VPS Mean Latency Total subscores. Cognitive flexibility latency was calculated as the average *z*-score from Anagrams Mean Latency (ms) and VPS Mean Latency Total (ms) subscores. Cognitive flexibility RCS was calculated using the total correct z-score divided by the sum of the latency score for Anagrams and VPS [31]. Memory was calculated as the average *z*-score from HVLT subscores and the REY-Delayed Copy Total Correct subscore. Interactive term η^2^ was used to describe effect sizes for MLM models, and effect sizes were quantified as small (η^2^ = 0.01), medium (η^2^ = 0.06), and large (η^2^ = 0.14) based on the recommend cutoffs [38,39]. HVLT = Hopkins Verbal Learning Test; REY-CFT = Rey–Osterrieth Complex Figure Test; VPS = Visuospatial Problem Solving; ms = milliseconds; FA = False Alarm; RCS = Rate Correct Score. * *p* < 0.05.

## Data Availability

Data can be requested by contacting the PI and corresponding author of the study (PI: Beversdorf).
